# Hepatitis B Virus DNA Polymerase Displays an Anti-Apoptotic Effect by Interacting with Elongation Factor-1 Alpha-2 in Hepatoma Cells

**DOI:** 10.4014/jmb.2002.02039

**Published:** 2020-10-30

**Authors:** Xianli Niu, Shirong Nong, Junyuan Gong, Xin Zhang, Hui Tang, Tianhong Zhou, Wei Li

**Affiliations:** 1 Department of Biochemistry and Molecular Biology, Zunyi Medical University, Zhuhai, Guangdong 5904, P.R. China; 2 Key Laboratory of Genetic Engineering and Medicine, Key Laboratory of Viral Biology, Jinan University, Guangzhou, Guangdong 51063, P.R. China

**Keywords:** Hepatitis B virus, chronic hepatitis B, HBV DNA polymerase, apoptosis, eEF1A2

## Abstract

Hepatitis B virus (HBV) genome P-encoded protein HBV DNA polymerase (Pol) has long been known as a reverse transcriptase during HBV replication. In this study, we investigated the impact of HBV Pol on host cellular processes, mainly apoptosis, and the underlying mechanisms. We showed a marked reduction in apoptotic rates in the HBV Pol-expressed HepG2 cells compared to controls. Moreover, a series of assays, *i.e.*, yeast two-hybrid, GST pull-down, co-immunoprecipitation, and confocal laser scanning microscopy, identified the host factor eEF1A2 to be associated with HBV Pol. Furthermore, knockdown of eEF1A2 gene by siRNA abrogated the HBV Pol-mediated anti-apoptotic effect with apoptosis induced by endoplasmatic reticulum (ER) stress-inducer thapsigargin (TG), thus suggesting that the host factor eEF1A2 is essential for HBV Pol’s anti-apoptosis properties. Our findings have revealed a novel role for HBV Pol in its modulation of apoptosis through integrating with eEF1A2.

## Introduction

Hepatitis B virus (HBV) infection is a major cause of chronic liver diseases, including chronic hepatitis, liver cirrhosis, and hepatocellular carcinoma (HCC), which is among the most common malignant tumors leading to death in countries of the Asia–Pacific region and beyond [1-4]. Indeed, a large proportion of HCC patients have a history of HBV infection, and the viral proteins encoded by the HBV genome are likely responsible for carcinogenesis transformation through interaction with the host factors [3, 5]. In recent decades, our understanding of the biological roles of virus-encoded proteins in the viral life cycle and pathogenesis of HBV-associated liver diseases (particularly liver cancer) has advanced. For example, HBV X protein (HBVx), which is encoded by the viral genome X with a calculated molecular weight of 17 kDa, participates in viral replication as a cofactor of viral transcription as well as in malignant transformation through modulating cell proliferation and programmed cell death by inducing or inhibiting apoptosis [6]. In comparison with the multiple roles for HBVx, HBV DNA polymerase (Pol), a viral genome P-encoded protein with a molecular mass weight of approximately 91 kDa, possesses transcriptase activity and functions as RNA-dependent DNA polymerase, which plays an essential role in HBV replication within a cytoplasmic nucleocapsid particle in which HBV core protein and the RNA template are recruited along with several host factors [7]. In the process of viral replication, the interaction of HBV Pol with heat shock protein 90 (Hsp90) initiates transcription and virion assembly [8]. In addition, some evidence has demonstrated that HBV Pol and eukaryotic initiation factor 4E (eIF4E) form a complex which is involved in the virus packaging. These studies have indicated that HBV Pol exerts its biological function through interaction with host protein factors either directly or indirectly.

In our own preliminary research, the host proteins that could interact with HBV Pol were screened and identified by immunoprecipitation (IP) in combination with liquid chromatography tandem mass spectrometry (LC-MS/MS) (data not shown). Among the 45 candidate host protein factors, eukaryotic translation elongation factors 1 alpha 2 (eEF1A2) ignited our interest in exploring a novel role for HBV Pol in the biological processes of host cells with a main focus on the apoptosis of hepatoma cells. Eukaryotic translation elongation factors 1 alpha (eEF1A), eEF1A1 and eEF1A2 are considered to play key roles in protein synthesis [9]. Furthermore, eEF1A1 is involved in regulating cell proliferation and cell death while eEF1A2 favors oncogenesis as demonstrated by evidence showing that overexpression of eEF1A2 resulted in malignant transformation in nude mice [10-12].

In the present paper, we documented an unexpected role for HBV Pol inhibiting apoptosis of hepatoma cells. To explore the associated molecular mechanisms in hepatoma cells, we examined and verified the interaction of HBV Pol with the host factor eEF1A2 by utilizing the following techniques: co-immunoprecipitation (Co-IP), yeast two-hybrid assay, and glutathione S transferase (GST) pull-down analysis. In parallel, we applied confocal laser scanning microscopy to validate the co-localization of the two proteins. Furthermore, we investigated the effects of eEF1A2 gene knockdown by siRNA as well as overexpression of eEF1A2 on the HBV Pol-mediated anti-apoptotic effect with apoptosis induced by the endoplasmic reticulum stress (ERS)-inducer thapsigargin (TG). Our results will help to shed new light on HBV Pol’s role in the pathogenesis of HBV-induced liver diseases, with the aim of providing novel therapeutic targets for more effective prevention, diagnosis, and treatment of HBV-related liver diseases.

## Materials and Methods

### Materials

HepG2 hepatoma cell line was obtained from the Department of Biochemistry and Molecular Biology of Jinan University (Guangzhou, China). HepG2 cells were maintained in Dulbecco’s Modified Eagle Medium (DMEM) supplemented with 10% (v/v) fetal bovine serum (FBS), ampicillin (100 units/ml), and streptomycin (100 μg/ml) at 37°C in a carbon dioxide (CO_2_) incubator containing 5% (v/v) CO_2_. All the other reagents were commercially available.

### Constructs, siRNA, and Transfection

We initially extracted the HBV genome RC-DNA from the HBV-positive serum and constructed the recombinant plasmid pcDNA3.1-HBV. All patients consented to participate in the experiments and the IRB number is GDJN-20190038. The HBV genome P open reading frame was subsequently inserted into the empty vectors pcDNA3.1-Flag and pGBK-T7 to construct the pcDNA3.1-Flag-Pol- and pGBK-T7-Pol-containing HBV genome P open reading frame (ORF). The eEF1A2 gene cDNA was amplified by PCR using HepG2 total cDNA. The recombinant plasmid pcDNA-His-eEF1A2 was constructed by immobilization of the eEF1A2 gene into the plasmid vector. We confirmed them using restriction enzyme digestion and gene sequencing. eEF1A2-specific siRNAs and non-specific control siRNAs were purchased from Takara Bio Inc. (Japan). For transfection, HepG2 cells were cultured to reach 50–80% confluence; cells were transfected or co-transfected with appropriate vectors using Lipofectamine 2000 according to the manufacturer’s protocol (Invitrogen, Thermo Fisher Scientific, USA).

### Real-Time Quantitative RT-PCR, Western Blots, and Co-Immunoprecipitation

Real-time quantitative RT-PCR, western blot analysis, and co-immuneprecipitation (Co-IP) were performed following the standard protocols in our laboratory.

### Yeast Two-Hybrid Assay

The yeast two-hybrid (Y2H) assay was performed to detect interaction between HBV Pol and eEF1A2. The pGBK-T7-Pol vector harboring GAL4 DNA-binding domain (BD) and HBV Pol was served as a bait vector while the pGAD-T7-eEF1A2 containing GAL4 activation domain (AD) and eEF1A2 was used as a prey vector. Both recombinant bait and prey vectors were co-transformed into AH109 yeast cells. Single colonies were selected and screened for growth on SD selection medium lacking tryptophan and leucine (SD/-Trp/-Leu) or lacking tryptophan, leucine, histidine, and adenine (SD/-Trp/-Leu/-His/-Ade). PGAD-T7-T and pGBK-T7-53 were used as positive controls, with pGAD-T7-T and pGBK-T7-Lam as negative controls while recombinant vectors pGAD-T7 and pGBK-T7-pol were also co-transformed into AH109 yeast cells as control for potential auto-activation of the prey protein. Colonies that turned blue indicated interaction of HBV Pol with eEF1A2.

### GST Pull-Down Assay

A GST pull-down assay was conducted to further examine the interaction of HBV Pol and host factor eEF1A2 in vitro. In brief, glutathione S-transferase (GST) or GST-eEF1A2 was induced by IPTG at 18°C with concentration of 0.2 mM, and SDS-PAGE electrophoresis was used to detect the expression of pGEX-4T-1-eEF1A2 recombinant bacteria in the Coomassie brilliant blue staining band around 76 kDa. The protein was immobilized onto GST beads at 4°C overnight. GST beads were added to the eukaryotic fusion protein, Parafilm sealed, placed on ice, and followed by incubation with the target protein for 6 h, Flag-Pol, which originated from Flag-Pol-expressed HepG2 cell lysates. After all unbound proteins were washed with washing buffer at least five times, the bound protein complex was eluted with elution buffer, and then analyzed by western blot to detect the target protein HBV Pol band (91 kDa). The GST protein and Flag-Pol protein were incubated as control group, and incubated with eEF1A2-GST protein and Flag-Pol protein as experimental group. The supernatant of the two groups was detected by western blot for the expression of Flag-Pol (Input).

### Immunofluorescence and Confocal Microscopy

To examine the subcellular localization of HBV Pol and eEF1A2 and to explore co-localization of the two proteins, HBV Pol and eEF1A2 proteins were transiently expressed in HepG2 cells for 48 h. The HepG2 cells were fixed in paraformaldehyde, rinsed briefly with PBS, and permeabilized with 0.1% (v/v) Triton X-100 for 5 min. The cells were subsequently incubated with primary antibodies for 60 min and washed at least three times with PBS, followed by application of secondary antibodies conjugated with fluorescent proteins.

Hoechst 33342 (Thermo Fisher Scientific, USA) nucleic acid staining, which emits blue fluorescence, was used for nuclear staining in fluorescence microscopy. Subcellular localizations of HBV Pol and eEF1A2 proteins were determined by fluorescence microscopy with GFP fluorescent signals captured with an excitation wave length of 488 nm and an emission wavelength of 505–530 nm, and with red fluorescent signals resulting from chlorophylls visualized at an emission wavelength of 650 nm.

### Flow Cytometric Analysis of Apoptosis of Hepatoma Cells

Annexin V-FITC/PI Apoptosis Detection Kit (Abcam, USA) was used to detect early- and late-stage apoptosis cells according to the manufacturer’s instructions.

### Statistical Analysis

Data from experiments in this study were expressed as mean ± SE. The significance between samples were determined using Student’s t-test. All experiments in the present study included at least triplicate samples for each treatment group. Representative results were also presented. *p* < 0.05 was considered significant and denoted by *.

## Results

### Overexpression of HBV DNA Polymerase Inhibits Apoptosis of HepG2 Cells

**Fig. 1:**
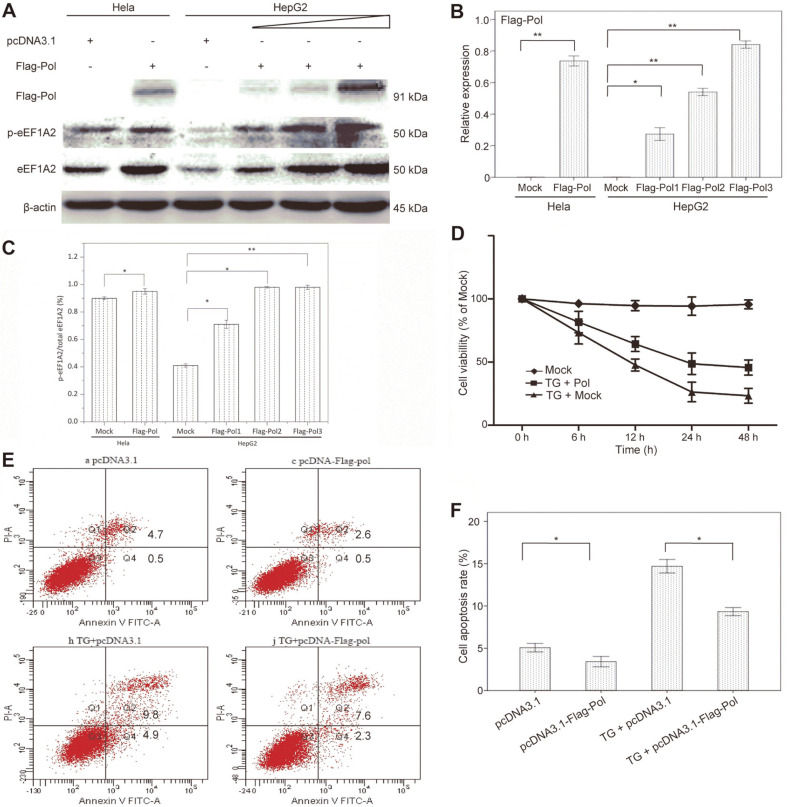
Overexpression of HBV DNA polymerase inhibits apoptosis of HepG2 cells. (**A**) Effects of overexpression of HBV Pol in HepG2 and Hela cells on the expression of eEF1A2 protein. (**B**) Effects of overexpression of HBV Pol in HepG2 and Hela cells on the relative expression of HBV Pol. (**C**) and (**D**) Effects of overexpression of HBV Pol in HepG2 and Hela cells on the relative expression of P- eEF1A2 and eEF1A2. (**E**) The cell viability of HepG2 cells under the overexpression of HBV Pol and eEF1A2. (**F**) Effect of HBV Pol on apoptosis of HepG2 cells with or without induction of endoplasmic reticulum (ER) stress by thapsigargin (TG). (**G**) Percentages of HepG2 cells undergoing apoptosis with or without ER stress inducer TG (**p < 0.01 and *p < 0.05 compared with mock-transfected cells).

We first determined whether HBV DNA Pol could affect cell viability and apoptosis of HepG2 and Hela cells. Overexpression of HBV Pol, which was successfully achieved by transfection of the HepG2 cells with pcDND3.1-Flag-Pol (Fig. 1A), resulted in an increase in cell survival rate (Fig. 1D) compared to control cells transfected with the parental vector. In parallel, using flow cytometry, we examined the effects of HBV Pol on apoptosis of HepG2 cells by using an Annexin V-FITC/PI Apoptosis Detection Kit (Abcam). As shown in Figs. 1E-1F, in conditions with or without induction of ERS by TG, apoptotic rates in the HBV Pol-expressed HepG2 cells significantly declined compared to the empty parental vector transfected cell, with a greater anti-apoptotic effect on the TG-induced HepG2 cells thus indicating that overexpression of HBV Pol inhibited apoptosis of HepG2 cells. According to previous research, overexpression of HBV DNA polymerase causes downregulation of Fas expression in HepG2 cells, and while the expression of anti-apoptotic genes Bcl-2 and Bcl-xl rises, the expression of pro-apoptotic genes Bax, Bak and Bad decreases. The significant decrease of apoptosis ratio in liver cancer cell reduces its killing sensitivity to NK cells, resulting in immune escape of HBV liver cancer. Furthermore, an enhancement of HBV Pol expression led to increases in levels of endogenous eEF1A2 and its phosphorylation (p-eEF1A2) (Figs. 1A-1C).

### Interaction between HBV DNA Polymerase and Host Factor eEF1A2

We next performed experiments to gain insight into the mechanism whereby HBV Pol exerts an anti-apoptotic effect in HepG2 cells. In our preliminary studies using the LC-MS/MS technology combined with bioinformatics, the host protein eEF1A2 was screened out to associate with HBV Pol. It is intriguing to consider that eEF1A2 could be involved in the HBV Pol-mediated anti-apoptotic action. Therefore, we examined and verified the interaction between HBV Pol and eEF1A2.

**Fig. 2:**
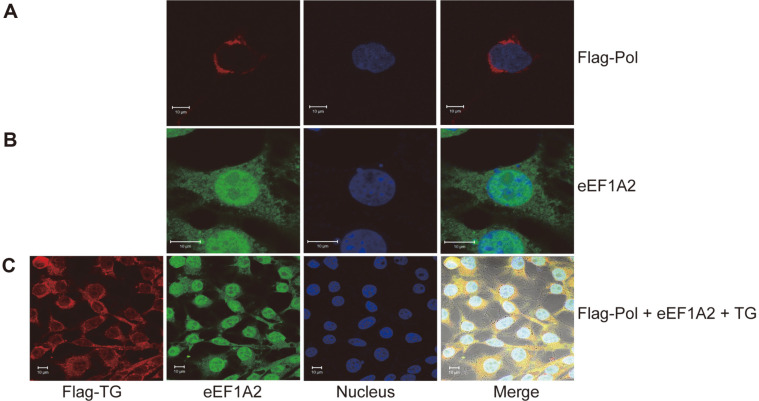
Subcellular co-localization of HBV Pol and eEF1A2 in HepG2 were visualized under a laser scanning confocal microscope. (**A**) Subcellular localization of HBV Pol in HepG2 transfected with pcDNA3.1-Flag-Pol. (**B**) Subcellular localization of eEF1A2 in HepG2 cells transfected with pcDNA3.1-His-eEF1A2. (**C**) Co-localization of HBV Pol and eEF1A2 in HepG2 co-transfected with pcDNA3.1-Flag-Pol and pcDNA3.1-His-eEF1A2.

To investigate the subcellular localization of HBV Pol and eEF1A2 proteins, immunofluorescence (IF) microscopy was performed after the HBV Pol protein was transiently expressed in the HepG2 cells for 48 h. As shown in Fig. 2, the Flag-tagged HBV Pol protein presented mainly in the cytoplasm with red fluorescent signals (Fig. 2A), while endogenous eEF1A2 expressed in both the nucleus and cytoplasm determined by green fluorescent signals (Fig. 2B). The co-localization of both proteins was observed in the cytoplasm without TG induction (Fig. 2C). Furthermore, HepG2 cells were co-transfected with the pcDNA3.1-Flag-Pol and pcDNA3.1-His-eEF1A2 and induced with TG. A merge of red and green fluorescence signals in the nucleus resulting from HBV Pol and eEF1A2 was visualized, indicating that co-localization of the two proteins in the nucleus was dramatically increased after TG induction. It is speculated that certain proteins in the nucleus might lose their functions after the TG treatment. We have added this in the revised manuscript.

**Fig. 3:**
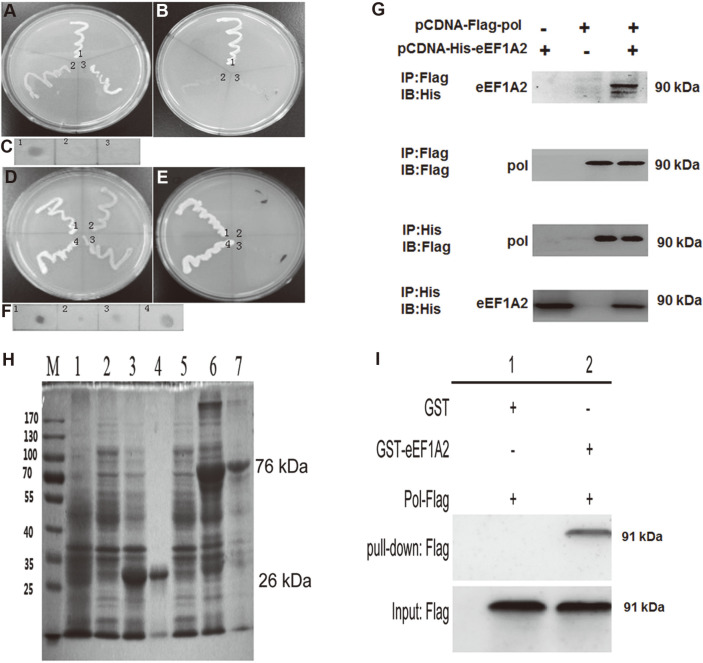
A Yeast two-hybrid analysis of in vitro interaction between HBV Pol and eEF1A2. (**A**) and (**D**) Selection and screen of single colonies for growth on SD selection medium lacking tryptophan and leucine (SD/-Trp/-Leu). (**B**) and (**E**) Selection and screen of single colonies for growth on SD selection medium lacking tryptophan, leucine, histidine, and adenine (SD/-Trp/-Leu/-His/-Ade). (**C**) and (**F**) Formation of the binding complex (lane 1) and (lane 4) was indicated by appearance of blue colonies in β-galactosidase assay. (**G**) Co-immunoprecipitation analysis of interaction between HBV Pol and eEF1A2 in HepG2 cells; GST pull-down revealed association of HBV Pol with eEF1A2 in vitro. (**H**) Expression and purification of fusion protein eEF1A2-GST. lane1: negative control, lane2: positive control, lane 3 total GST, lane 4: purified GST, lane 5 total pGEX- 4T-1, lane 6: total eEF1A2, lane 7: purified eEF1A2. (**I**) GST pull-down analysis of in vitro interaction of HBV Pol with eEF1A2.

Yeast two-hybrid (Y2H) analysis was also conducted to examine the interaction between HBV Pol with eEF1A2. As pGBKT7-Lam and pGADT7-T-transformed yeast AH109 strain (negative control) could not grow on SD selection medium lacking adenine, histidine, leucine, and tryptophan (SD/–Ade/–His/–Leu/–Trp) (Lane 2 in Figs. 3B and 3E) but was capable of growing on SD selection medium lacking leucine and tryptophan (SD/–Leu/–Trp) (Lane 2 in Figs. 3A and 3D), this suggested the feasibility of the Y2H system (Lane 2 in Figs. 3A, 3B, 3D, and 3E). Yeast AH109 strain transformed with pGBKT7-pol and pGADT7 (Lane 3 in Figs. 3D and 3E) and displayed the same growing states as the above negative control (Lane 2 in Figs. 3D and 3E), thus indicating that no auto-activation of the prey protein occurred, the same as the pGBKT7 and pGADT7-eEF1A2 to exclude the auto-activation of the prey (Lane 3 in Figs.3A and 3B). However, yeast AH109 cells (transformed with pGBKT7-pol and pGADT7-eEF1A2) showed the ability to grow on both selection (SD/–Ade/–His/–Leu/–Trp) and (SD/–Leu/–Trp) as observed in the positive control strain harboring recombinant bait and prey vectors pGBKT7-p53 and pGADT7-T (Lane 1 in Figs. 3A, 3B, 3D, and 3E), indicating interaction between HBV Pol and eEF1A2 in vitro (Lane 4 in Figs. 3D and 3E). The interaction of HBV Pol and eEF1A2 was also demonstrated in the α-galactosidase (α-gal) assay by the appearance of blue colonies due to formation of the binding complex HBV Pol-eEF1A2 (Figs. 3C and 3F).

Specifically, we performed Co-IP analysis to examine the interaction between HBV Pol (pcDNA3.1-Flag-Pol or Pol-wild type) and eEF1A2 (pcDNA3.1-His-eEF1A2) using empty vector as negative control. Cell lysates were prepared and subjected to IP of Flag-Pol with anti-Flag antibody. As shown in Fig. 3G, Flag-Pol expression was detected in the three groups of cell lysis supernatant (Input), then feasibility of the Co-IP system was validated by detection of HBV Flag-Pol and pcDNA3.1-His-eEF1A2. An immunoblotting (IB) analysis of the immunoprecipitates with anti-Flag specific antibody revealed His-tagged eEF1A2, thus suggesting the association between HBV Pol and host factor eEF1A2 in the HepG2 cells transiently transfected with pcDNA3.1-His-eEF1A2 and pcDNA3.1-Flag-Pol. The His-eEF1A2 fusion protein at 50 kDa was detected in the supernatant and Co-IP results of the experimental group. In contrast, His-tagged eEF1A2 was not detectable under control condition. The Co-IP results suggest that HBV Pol interacted with the host factor eEF1A2.

The GST pull-down assay was then carried out to further examine the interaction between HBV Pol and host factor eEF1A2 in vitro. As shown in Figs. 3H and 3I, DS-PAGE electrophoresis showed that GST or Gst-eEF1A2 fusion protein induced by IPTG at 18oC was expressed in the supernatant of the bacteria. HBV Pol protein was detectable in GST pull-down analysis with the fusion protein eEF1A2-GST but not with GST alone in western blot, thus demonstrating the interaction of HBV Pol with eEF1A2 in vitro.

### Effects of eEF1A2 on HBV Pol-Associated Anti-Apoptotic Effect

**Fig. 4:**
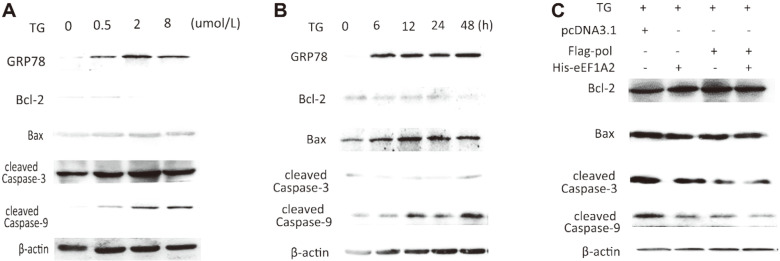
Effects of eEF1A2 on HBV Pol-associated anti-apoptotic effect by examination of selected apoptosisrelated factors after apoptosis induced by TG. (**A**) Effects of the doses of TG alone on levels of selected apoptosis-related protein factors. (**B**) Time-dependent effects of TG alone on levels of apoptosis-related protein factors. (**C**) Effects of eEF1A2 on HBV Pol-associated anti-apoptotic effect.

Next, we determined the protein levels of the apoptosis-related factors, including GRP78, Bcl2/Bax, cleaved caspase3, caspase9, with apoptosis induced by TG (Figs. 4A and 4B). As expected, the protein levels of GRP78, Bax, and cleaved protein Caspase9/3 increased while anti-apoptotic factor Bcl-2 decreased in response to both concentration and exposure duration with TG (Figs. 4A and 4B). Furthermore, overexpression of HBV Pol and eEF1A2 in the cells treated with 2 umol/l of TG for 24 h resulted in decreases in levels of GRP78, Bax, cleaved Caspase9/3, and other pro-apoptotic factors. However, increases in levels of the anti-apoptotic Bcl-2 protein compared with the control group (Fig. 4C). These findings suggested the anti-apoptotic effects of HBV Pol and eEF1A2.

**Fig. 5:**
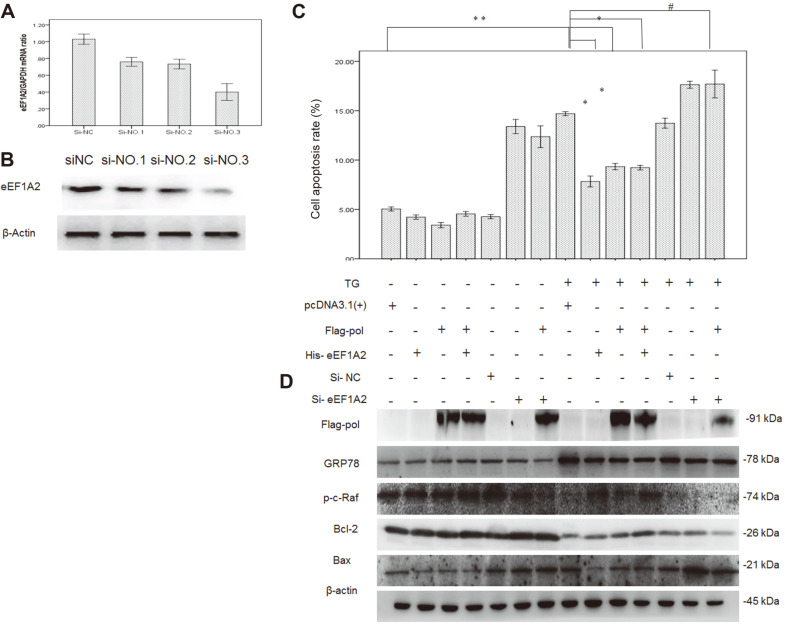
Effects of eEF1A2 on HBV Pol-associated anti-apoptotic effect. (**A**) Real-time qRT-PCR analysis of the effects of eEF1A2-siRNA on eEF1A2 mRNA level. (**B**) Western blot analysis of the effects of eEF1A2-siRNA on eEF1A2 protein level. (**C**) Statistical analysis of apoptotic rate in HepG2 cells by flow cytometry. (**D**) Western blot analysis of the effects of eEF1A on the HBV Pol-associated anti-apoptosis in HepG2 cells. **p < 0.01 compared with pcDNA 3.1-transfected cells; ^#^*p* > 0.05 and *p < 0.05 compared with pcDNA3.1 (TG) transfected cells.

Based upon the above extensive experiments, which demonstrated a novel biological function for HBV Pol to inhibit apoptosis and the interaction of HBV Pol with the host factor eEF1A2, we finally investigated if eEF1A2 could play a role in the HBV Pol-associated anti-apoptotic effect. This hypothesis was then tested by knockdown and overexpression of the eEF1A2 gene in HepG2 cells with ERS by TG. Fluorescence quantitative PCR analysis was used to determine the interference efficiency of eEF1A2 siRNA. As a result, the si-NO.3 showed the most effective interference with the efficiency over 60%. As expected, silencing of the eEF1A2 gene with eEF1A2-specific siRNA markedly brought down the levels of eEF1A2 mRNA (Fig. 5A) and protein (Fig. 5B). The apoptosis assay results showed that HBV Pol significantly inhibited apoptosis of HepG2 cells compared with control. However, overexpression of HBV Pol did not alter apoptotic rates in eEF1A2, which was abrogated by eEF1A2-siRNA. These findings suggested that the host factor eEF1A2 participated in HBV Pol-mediated anti-apoptotic effects compared with controls, and that HBV Pol and eEF1A2 may share a similar pathway, through which apoptosis of HepG2 cells were inhibited by both proteins, while HepG2 cells were insensitive to anti-apoptotic effects of HBV Pol after knockout of eEF1A2. As shown in Fig. 5C, comparison of cell apoptosis rate was detected by flow cytometry in different treatment groups of HepG2 cells. In parallel, the effect of eEF1A2 on HBV Pol-associated anti-apoptotic effect was further evaluated by western blot analysis of apoptosis-related proteins (Fig. 5D), with decrease in the phosphorylation level of c-Raf (p-c-Raf) and Bcl2 and increase in Bax and GRP78 protein in response to knockdown of the eEF1A2 gene in HepG2 cells with TG induction, compared with the control group.

## Discussion

In the present study, we identified a novel role for HBV Pol in its modulation of apoptosis in hepatoma cells. Our major novel findings were as follows: (1) A marked reduction in apoptotic rates were seen in HBV Pol-expressed HepG2 cells suggesting an anti-apoptosis property of HBV Pol. (2) The host factor eEF1A2 was found to be associated with HBV Pol, and the interaction of the two proteins was verified by a set of assays: Co-PI, yeast two-hybrid, GST pull-down, and confocal laser scanning microscopy; (3) Silencing of eEF1A2 gene by siRNA abrogated the HBV Pol-mediated anti-apoptotic effect in hepatoma cells, indicating the role for eEF1A2 in the HBV Pol-induced anti-apoptosis of hepatoma cells; (4) Bax is a water-soluble related protein homologous to Bcl-2. It is an apoptosis-promoting gene in the Bcl-2 gene family. Overexpression of Bax can antagonize the protective effect of Bcl-2 and cause cell death. Currently, it is often used in tumor research with BCL-2. The Bax gene is the most important apoptotic gene in the human body and belongs to the Bcl-2 gene family. The encoded Bax protein can form a heterodimer with Bcl-2 and resulted in a depressing effect. Previous studies have found that the ratio of Bax/Bcl-2 proteins is a key factor in determining the strength of apoptosis inhibition. Therefore, it is believed that Bax is one of the most important apoptosis-promoting genes. The anti-apoptosis effect of HBV Pol may induce the expression level of Bcl2/ bax protein by activating c-Raf pathway through *eEF1A2* gene.

HBV Pol is considered responsible for RNA-directed DNA synthesis in the HBV life cycle. Our results identified an unusual role of HBV Pol in preventing HepG2 cells from apoptosis, thus leading to impaired apoptosis of hepatoma cells. Apoptosis, like cell proliferation and other cellular processes, is highly modulated for the maintenance of tissue homeostasis. A wealth of scientific evidence has demonstrated that unbalanced apoptosis results in malignancy transformation and even different forms of cancer [13, 14]. HBV Pol’s anti-apoptotic action as well as other viral proteins could contribute to HBV-induced hepatocellular carcinogenesis, and could explain, to some degree, the clinical observation in which approximately 50% of patients with liver cancer also suffered from persistent HBV infection. Furthermore, our results obtained by knockdown of the eEF1A2 gene in HBV Pol-expressed HepG2 cells demonstrated an association of HBV Pol with the host factor eEF1A2, which is probably involved in the HBV Pol-mediated-impaired apoptosis of HepG2 cells. In contrast, with the expression of HBV Pol cells, after knockout of eEF1A2, the cell apoptosis rate increased compared with the control group (Fig. 5C), indicating that eEF1A2 could mediate the anti-apoptosis mechanism of HBV Pol. When knockout of eEF1A2, HepG2 cells were not sensitive to the anti-apoptosis function of HBV Pol with TG induction.

eEF1A2, one of the isoforms of eEF1A, is a multifunctional protein with versatile roles in not only protein synthesis but also other cellular processes including cytoskeletal movement, signal transduction, viral replication, cell cycle regulation, and apoptosis [15-18]. Research has also shown that silencing of the eEF1A2 gene decreases cell proliferation and increases the apoptosis rates in hepatoma cells [19, 20]. In addition, eEF1A2 is upregulated in 43% of HCC, and overexpression of eEF1A2 in gemcitabine-treated cancer cells resulted in enhancement of phosphorylated Akt and cell viability, thus suggesting that eEF1A2 possesses ability to activate the Akt pathway, which has been shown to be activated in approximately 50% of primary hepatocellular carcinoma cases [21]. These findings have supported the fact that eEF1A2 exerts its oncogenic behavior by participating in the regulation of the P13K pathway. In addition, eEF1A2 has an antiapoptotic effect, and eEF1A2 directly interacts with the antioxidant peroxiredoxin thus protecting cells from oxidative stress-induced apoptosis by regulating the Bcl2 [22, 23]. Bcl-2 protein is the coding product of Bcl-2 proto-oncogene and is a cell survival-promoting factor. It is a membrane integral protein with a molecular weight of 26 kDa. It is located in the mitochondria, endoplasmic reticulum and continuous perinuclear membrane, and is widely expressed in the embryonic tissues. The Bcl-2 protein family is a special family and 25 Bcl-2 family homologous proteins have been found. Some of its members promote apoptosis, such as Bax. And some members prevent cell apoptosis, such as Bcl-2, Bcl-x, Bcl-w. Overexpression of Bcl-2 can antagonize the protective effect of Bax and demonstrate anti-apoptosis effects.

When host cells are infected with HBV, the HBV large surface antigen overexpresses, resulting in protein accumulation in the endoplasmic reticulum, so that cells develop ERS [24, 25]. Then, the contents of Ca^2+^ in the endoplasmic reticulum are decreased and the Ca^2+^ equilibrium steady state is destroyed by TG, which results in dysfunction of the endoplasmic reticulum and induced ERS (endoplasmic reticulum stress). When the ERS occurs too long, unfolded protein and misfolding protein accumulate leading to endoplasmic reticulum dysfunction, cell endoplasmic reticulum-related apoptosis initiation, and a large number of pro-apoptotic proteins become activated to induce cell apoptosis [26]. In the early stages of ERS, the rapid increase in GRP78 expression may be associated with the accumulation of protein that causes cell damage [27, 28]. GRP78 is a molecular chaperone in the endoplasmic reticulum, involved in the folding, transport and glycosylation of proteins [29]. eEF1A2 overexpression and knockout affects GRP78 protein expression levels, suggesting that eEF1A2 may be related to the regulation of ERS.

We found that overexpression of the eEF1A2 gene upregulated the c-Raf-1 protein expression and enhanced the levels of its phosphorylation. After silencing of the eEF1A2 gene by siRNA, c-Raf-1 protein expression and levels of its phosphorylated form declined (Fig. 5D). Upregulation of c-Raf-1 has been observed in HBV infection, liver cirrhosis, and hepatocellular carcinoma [25, 30]. The activated form of phosphorylation of c-Raf-1 represents a link between Ras and the MEK-ERK kinases; the phosphorylation cascade Raf-MEK-ERK is linear and the signaling pathway is involved in modulation of proliferation and apoptosis [11, 12]. Activated c-Raf is able to interact with Bcl-2 dimer complex, that transfers to mitochondria, and c-Raf is liberated from Bcl-2 by phosphorylation of Bad, inducing activation of Bcl-2 anti-apoptotic function [31]. Bcl-2 plays an important role in cell proliferation and apoptosis and Sanges *et al*. reported that eEF1A2 interacts with c-Raf enzymes and is capable of phosphorylation with c-Raf enzymes [10, 12]. eEF1A2 is associated with protein folding, promoting its proper folding and playing an important role in maintaining the stability of intracellular environment. Overexpression of eEF1A2 may reduce the protein accumulation under the ERS by TG, increase the Bcl2 protein, and then downgrade the GRP78. After knockout of eEF1A2, the Bcl2 protein is downregulated, the ERS and the proapoptotic factor Bax protein are increased, and cells end up in apoptosis. GRP78 may be resistant to adverse factors and up. When the endoplasmic reticulum stress occurs, eEF1A2 may be recruited to participate in this process, reduce the damage caused by ERS to cells, and then adjust the GRP78, Bcl2 protein levels, and improve cell survival rate. The anti-apoptotic effect of HBV Pol may be related to the level of Bcl2/Bax protein mediated by eEF1A2, and the anti-apoptotic ability of HBV Pol is decreased after knockout of eEF1A2. Thus, we postulated that HBV Pol’s association with eEF1A2 could activate the c-Raf-MEK-ERK signaling pathway, through which HBV Pol-expressed HepG2 cells display resistance to apoptosis.

Our study has some limitations. Although lines of evidence have pointed out the interaction of HBV Pol with eEF1A2, we could not include additional information about the binding sites of the two proteins and the action mode in three dimensions. As HBV Pol possesses a few functional domains, terminal protein (TP), DNA polymerase/reverse transcriptase, RNHase, and a non-essential spacer region which does not directly affect the enzymatic activities of HBV Pol [32], we could not exclude the possibility of whether the non-essential spacer region could be involved in the HBV Pol-mediated anti-apoptosis of hepatoma cells. We are not sure if it affects carcinogenesis from primary hepatocyte. With our longstanding interests in the field of HBV and its related diseases, further efforts are being made to pinpoint additional molecular mechanisms that illustrate how HBV Pol could recruit eEF1A2 along with its consequential impact, both on the HBV viral life cycle and HBV-related diseases. Since a cell culture model cannot replicate HBV in animals and humans, investigations using animal models and clinical samples of human subjects are also currently underway in our laboratory.

In summary, these findings suggest a novel role of HBV Pol in the apoptosis pathway under ERS induced with TG through eEF1A2. The molecules involved in the signaling pathways may represent better candidate targets in the prevention, diagnosis, and treatment of HBV-related liver diseases.
